# Diet-induced obesity in animal models: points to consider and influence on metabolic markers

**DOI:** 10.1186/s13098-021-00647-2

**Published:** 2021-03-18

**Authors:** Mariana de Moura e Dias, Sandra Aparecida dos Reis, Lisiane Lopes da Conceição, Catarina Maria Nogueira de Oliveira Sediyama, Solange Silveira Pereira, Leandro Licursi de Oliveira, Maria do Carmo Gouveia Peluzio, J. Alfredo Martinez, Fermín Ignacio Milagro

**Affiliations:** 1 Department of Nutrition and Health, Universidade Federal de Viçosa, Viçosa, Brazil; 2 Department of Nursing and Medicine, Universidade Federal de Viçosa, Viçosa, Brazil; 3 Department of General Biology, Universidade Federal de Viçosa, Viçosa, Brazil; 4 Department of Nutrition, Food Science and Physiology, Center for Nutrition Research, University of Navarra, Pamplona, Spain; 5 Centro de Investigación Biomédica en Red de La Fisiopatología de La Obesidad Y Nutrición (CIBERobn), Carlos III Health Institute, Madrid, Spain; 6 IdiSNA, Navarra Institute for Health Research, Pamplona, Spain; 7 Madrid Institute of Advanced Studies (IMDEA Food), Food Institute, Madrid, Spain

**Keywords:** Obesity, High-fat diet, Obesogenic diet, Animal model, Inflammation

## Abstract

Overweight and obesity are a worldwide public health problem. Obesity prevalence has increased considerably, which indicates the need for more studies to better understand these diseases and related complications. Diet induced-obesity (DIO) animal models can reproduce human overweight and obesity, and there are many protocols used to lead to excess fat deposition. So, the purpose of this review was to identify the key points for the induction of obesity through diet, as well as identifying which are the necessary endpoints to be achieved when inducing fat gain. For this, we reviewed the literature in the last 6 years, looking for original articles that aimed to induce obesity through the diet. All articles evaluated should have a control group, in order to verify the results found, and had worked with Sprague–Dawley and Wistar rats, or with C57BL-/-6 mice strain. Articles that induced obesity by other methods, such as genetic manipulation, surgery, or drugs were excluded, since our main objective was to identify key points for the induction of obesity through diet. Articles in humans, in cell culture, in non-rodent animals, as well as review articles, articles that did not have obesity induction and book chapters were also excluded. Body weight and fat gain, as well as determinants related to inflammation, hormonal concentration, blood glycemia, lipid profile, and liver health, must be evaluated together to better determination of the development of obesity. In addition, to select the best model in each circumstance, it should be considered that each breed and sex respond differently to diet-induced obesity. The composition of the diet and calorie overconsumption are also relevant to the development of obesity. Finally, it is important that a non-obese control group is included in the experimental design.

## Introduction

Obesity is a global public health issue with high prevalence in all age groups [1, 2]. It generates a considerable social and economic impact, since it affects people’s health and quality of life [2]. Classically, obesity is defined as a visceral and subcutaneous lipid accumulation and body weight gain that may impair health [3, 4]. However, it is frequent to be accompanied by the deposition of lipids (ectopic fat) in non-adipose tissues, such as the liver [5].

The treatment and prevention of obesity involves the control of body weight and adiposity through a negative energy balance in which both, diet and physical activity, are important. But, due to changes in people's lifestyles, with less physical activity and shifts in eating behavior, the study of alternatives for the treatment of obesity, such as functional foods, and bioactive compounds, is gaining increasing relevance [2].

Obesity rates are increasingly higher [1, 2], which indicates that the strategies currently used are insufficient to control this disease, and that preclinical studies with this disease are still necessary [6]. To study the development of obesity and its risk factors, researchers use diet-induced obesity animal models, since these models reproduce with greater reliability human obesity in comparison with genetic models [7]. In addition, studies with animal models are carried out under controlled conditions, which facilitates the understanding of the results.

This article aims to evaluate diets-induced obesity models in mouse and rat published in the last 6 years. It seeks to identify which are the main methodological strategies to induce obesity through diet, as well as identifying which are the main parameters to be taken into account to achieve a successful model.

## Methodology

The search for articles was carried out manually on PubMed database by a single researcher in February 2020. The combination of the descriptors used was “*diet*” + “*obesity*” and “*high fat diet*” + “*obesity*”.

In the PubMed database the following filters were selected: “species/other animals”, “case reports”, “clinical trial”, “clinical trial veterinary”, “comparative study”, “controlled clinical trial”, “evaluation study”, “newspaper article”, “observational study”, “observational study veterinary”, “periodical index”, “programmatic clinical trial”, “randomized controlled trial”, “twin study” e “validation study”. Duplicated articles were excluded and the rest were evaluated according to the inclusion and exclusion criteria (Table 1). We want to highlight that the aim of this study was to evaluate the induction of obesity through the diet. Therefore, any study that used other ways to induce obesity was excluded. We also highlight that we evaluated only original articles, published between 2015 and 2020, in English, and that worked with Sprague–Dawley and Wistar rats, or with C57BL-/-6 mice strain.

**Table 1:** Inclusion and exclusion criteria used to evaluate the pre-selected articles

Inclusion	Exclusion
Diet-induced obesity The main objective was the induction and evaluation of obesity Study must be done with Sprague–Dawley or Wistar rats, or with C57BL-/-6 mice strain Original articles Presence of a control group Used commercial diets or produced them from standard ingredients Published in the last 6 years (2015–2020) Articles in English	Genetic manipulation Drug-induced obesity Surgically induced obesity Main objective was the induction and/or evaluation of other diseases affect for obesity (diabetes, metabolic syndrome, heart disease, liver disease, dyslipidemia and surgery for weight loss) Main objective was the induction and/or evaluation of other diseases (neurological diseases, cancer, rheumatological diseases, endocrine diseases, gynecological diseases and kidney diseases) The objective was to evaluate weight loss Studies in which there was no induction of obesity Studies in which there was an intervention before the obesity induction period Study on pregnancy and/or lactation model Study of a smoking model Study with humans Study with cell culture Study with non-rodent animals (ex: dogs, cats, birds, monkeys, rabbits …) Review articles, letters to the reviewer and book chapters Studies published in languages other than English

## Results and discussion

### Selection of articles

**Fig. 1:**
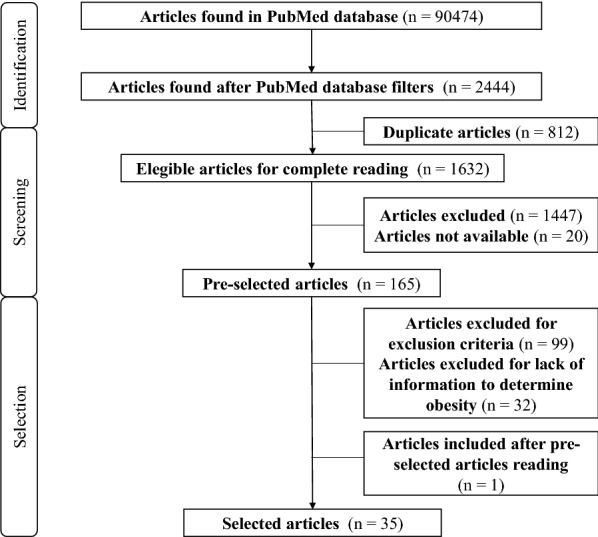
Flowchart for selecting articles

Initially, 90474 articles were found: 70658 by using the terms “*diet*” + “*obesity*” and 19816 by the terms “*high fat diet*” + “*obesity*”. After using the filters on PubMed database, 1625 articles were found for the “*diet*” + “*obesity*” search and 819 articles for the “*high fat diet*” + “*obesity*” search, totaling 2444 articles. After the exclusion of 812 articles that were duplicated, 1632 articles were considered eligible for reading titles and abstracts. According to the inclusion and exclusion criteria (Table 1), 165 articles were selected for full reading, 1447 articles were excluded and 20 articles were not available for reading, due to restricted access to their abstracts (Fig. 1).

After reading the complete articles, 99 were considered ineligible by some of the exclusion criteria established (Table 1). Another 14 articles were excluded because they did not include a control group and 18 articles were excluded because they did not provide enough information to conclude that the treatment led to obesity. After reading the selected articles, one additional article was included in the study, totaling 35 articles (Fig. 1).

Studies that used non-commercial diets, such as cafeteria diets, were excluded, since the nutritional composition varied widely compared to diets produced from standardized ingredients and commercial diets.

Processed foods can contain food additives and be low in vitamins and minerals, which can influence the composition of the intestinal microbiota and, consequently, the occurrence of obesity and other metabolic changes. So, in these cases, it is difficult to determine whether a metabolic outcome is only due to the high content of lipids or whether the high amount of food additives or low content of micronutrientes may influence it. In addition, diets produced from food may contain food additives, which make it difficult to assess the real effect of nutrients on the development of obesity [8].

### Diet composition

High-fat diets are commonly used to induce obesity in animals [8–10] since they generate adverse metabolic effects, meaning that diet is one of the major contributors to the obesity epidemic [1, 11].

All 35 studies evaluated used a high-fat diet to induce obesity; however, the amount of calories from lipids ranged from 41 to 60% (Table 2). Despite looking like a wide margin, according to Research Diets Inc [14], diet induced-obesity (DIO) animal models usually provides between 45 to 60% of calories from fats; therefore, all selected studies follow this recommendation. Nine studies [15–23] did not provide the composition of the macronutrients directly, which made it difficult to calculate the amount of calories from fat.

**Table 2:** Characterization of the experimental design of the evaluated studies

Article ID	Obesogenic diet			Control diet			Diet offering method	Intervention time	Animals		
	Type	%	kcal/g	Type	%	kcal/g			Type	Sex	Weeks-old
Asokan et al. 2018 [56]	High-fat^◊^	60.0 fat	–	Standard	–	–	Ad libitum	10 weeks	C57BL/6J mice	Male	6
Bortolin et al. 2018 [8]	High fat^◊^	60.0 fat	–	Control	–	–	Ad libitum	18 weeks	Wistar rats	Male	8
	Western^◊^	42.5 fat	–								
Heo et al. 2018 [2]	High-fat^◊^	60.0 fat, 20.0 carb., 20.0 protein	–	Low-fat^◊^	10.0 fat, 70.0 carb., 20.0 protein	–	Free access	10 weeks	Sprague–Dawley rats	Male	5
Hira et al. 2018 [24]	High-fat high-sucrose ▪	30.0 fat, 40.0 sucrose	5.1	Control (AIN-93G)	–	4.0	Free access	8 days	Sprague–Dawley rats	Male	5
								8 weeks			
Lee et al. 2018 [27]	High-fat B-MLCT ▪	30.0 fat	5.5	Low-fat B-MLCT ▪	7.0 fat	4.0	Ad libitum	16 weeks	C57BL/6J mice	Male	6
	High-fat E-MLCT ▪			Low-fat E-MLCT ▪							
	High-fat C-MLCT ▪			Low-fat C-MLCT ▪							
Iñiguez et al. 2018 [25]	High-fat^◊^	60.0 fat	–	Control	–	–	Pair-feeding	10 weeks	C57BL/6J mice	Male	8
Kazeminasab et al. 2018 [12]	High-fat^◊^	45.0 fat, 35.0 carb., 20.0 protein	–	Low-fat^◊^	10.0 fat, 70.0 carb., 20.0 protein	–	Ad libitum	12 weeks	C57BL/6J mice	Male	6
Matias et al. 2018 [3]	High-sugar^□^	25.9 fat, 52.3 carb., 24.8 protein	3.6	Control^□^	25.6 fat, 52.3 carb., 21.8 protein	3.5	Free access	20 weeks	Wistar rats	Male	4
	High-fat^□^	37.6 fat, 44.6 carb., 17.8 protein	4.6								
	High-fat/ high-sugar^□^	37.4 fat, 43.4 carb., 19.2 protein	4.5								
Miranda et al. 2018 [19]	High-fat	–	4.6	Control	-	3.7	Free access	6 weeks	Sprague–Dawley rats	Male	-
									Wistar rats	Male	-
Rocha Rodrigues et al. 2018 [57]	High-fat^◊^	71.0 fat, 11.0 carb., 18.0 protein	–	Standard^◊^	35.0 fat, 47.0 carb., 18.0 protein	-	Pair-fed	17 weeks	Sprague–Dawley rats	Male	6
Son et al. 2018 [21]	High-fat	–	–	Normal (AIN-93)	–	–	Ad libitum	8 weeks	Sprague–Dawley rats	Male	5
Virto et al. 2018 [62]	High-fat^●^	35.2 fat, 35.5 carb., 20.4 protein	5.4	Control^●^	3.0 fat, 60.0 carb., 16.0 protein	2.9	Ad libitum	26 weeks	Wistar rats	–	–
Wu et al. 2018 [30]	High-fat^◊^	23.7 fat, 53.3 carb., 23.0 protein	5.1	Normal^○^	13.3 saturated fat, 60.5 carb., 26.2 protein	3.6	Ad libitum	6 weeks	C57BL/6 mice	Male	6
Yuan et al. 2018 [35]	High fat^◊^	45.0 fat, 35.0 carb., 20.0 protein	–	Normal^◊^	4.0 fat, 62.0 carb., 18.0 protein	-	Ad libitum	18 weeks	C57BL/6J mice	Male	7
								24 weeks			
Aslani et al. 2017 [63]	High-fat^◊^	42.0 fat, 39.0 carb., 19.0 protein	–	Normal^◊^	11.0 fat, 61.0 carb., 28.0 protein	–	Ad libitum	12 weeks	Wistar rats	Male	8
Blancas-Velazquez et al.2017 [15]	High-fat high-sugar	–	–	Control^◊^	12.0 fat, 65.0 carb., 23.0 protein	2.8	Ad libitum	6 weeks	C57BL/6J mice	Male	6–8
Karimi et al. 2017 [18]	High-fat (AIN-76A)	–	5.5	Standard	–	3.8	Ad libitum	12 weeks	Sprague–Dawley rats	Male	6
								27 weeks			
Kim et al. 2017 [33]	High-fat^◊^	60.0 fat	–	Standard^◊^	12.0 fat, 64.0 carb., 24.0 protein	–	Ad libitum	6 weeks	C57BL/6 mice	Male	4
Kus et al. 2017 [68]	High-fat^◊^	60.0 fat	–	Control (AIN-93G)	–	–	–	15 weeks	C57BL/6 mice	Male	–
La Frano et al. 2017 [26]	High-fat high-cholesterol^◊^	60.0 fat	5.2	Low-fat low-cholesterol^◊^	10.0 fat	3.8	Ad libitum	18 weeks	C57BL/6J mice	Male	12
Pan et al. 2017 [61]	High-fat^◊^	45.0 fat	–	Normal^◊^	15.0 fat	–	Free access	16 weeks	C57BL/6J mice	Male	4
Picklo et al. 2017 [10]	High-fat oleic^◊^	50.0 fat, 30.0 carb., 20.0 protein	5.5	Low fat^◊^	16.0 fat, 64.0 carb., 20.0 protein	4.3	–	8 weeks	C57BL/6 mice	Male	12
	High-fat saturated^◊^										
Yang et al. 2017 [22]	High-fat	–	–	Control	–	–	–	6 weeks	Sprague–Dawley rats	–	–
Choi et al. 2016 [9]	High-fat^◊^	40.0 fat	–	Normal^◊^ (AIN-76)	11.5 fat	–	–	12 weeks	C57BL/6J mice	Male	8
Jambocus et al. 2016 [17]	High-saturated fat	–	–	Normal	–	–	Ad libitum	12 weeks	Sprague–Dawley rats	Male	3
Krishna et al. 2016 [1]	High-fat^◊^	41.0 fat, 40.0 carb., 19.0 protein	–	Standard^◊^	11.0 fat, 65.0 carb., 19.0 protein	–	Ad libitum	14 weeks	C57BL/6J mice	Male	3
Naidu et al. 2016 [20]	High-fat	–	–	Normal (AIN-93)	–	–	–	30 days	Wistar rats	Male	-
Zhao et al. 2016 [64]	High-fat^◊^	45.0 fat, 36.0 carb., 19.0 protein	–	Control^◊^	12.0 fat, 60.5 carb., 27.5 protein	–	–	8 weeks	Sprague–Dawley rats	Male	-
Huang et al. 2015 [16]	High-fat	–	–	Normal	–	–	Ad libitum	12 weeks	Sprague–Dawley rats	Male	–
Li et al. 2015 [51]	High-fat^●^	45.0 fat	–	Control^●^	10.0 fat	–	Ad libitum	22 weeks	C57BL/6J mice	Female	6–8
Nam et al. 2015 [28]	High-fat^◊^	45.2 fat, 38.0 carb., 16.8 protein	4.7	Low-fat^◊^	11.7 fat, 67.5 carb., 20.8 protein	3.8	Ad libitum	11 weeks	C57BL/6J mice	Male	4
Rodríguez- Rodríguez et al. 2015 [29]	High-fat^◊^	45.2 fat, 34.2 carb., 20.6 protein	–	Control^◊^	12.0 fat, 67.4 carb., 20.6 protein	–	Free access	12 weeks	C57BL/6NCrl mice	Male	8
Savetsky et al. 2015 [34]	High-fat^◊^	60.0 fat	–	Normal^◊^	13.0 fat	–	Ad libitum	10–12 weeks	C57BL/6J mice	Male	6
Wyatt et al. 2015 [13]	High-fat^◊^	60.0 fat	–	Control^◊^	10.0 fat	–	–	5 weeks	C57BL/6J mice	Male	6
								8 weeks			
Zhang et al. 2015 [23]	High-fat	–	–	Normal (AIN-93 M)	–	–	Ad libitum	16 weeks	C57BL/6 mice	Male	5

^□^, % g/kg; ▪, % w/w; ^◊^, % kcal; ^○^, % g/100 g; ^●^, Not determine the unit of %; B-MLCT, Non-enzymatically interesterified MLCT; E-MLCT, Enzymatically interesterified MCLT; C-MLCT, Commercially MLCT; carb., carbohydrate

The consumption of diets rich in fat can result in the development of human-like obesity, since it increases body adiposity and leptin, and stimulates the development of hypertension and glucose intolerance. Matias et al. [3] observed that offering a diet rich in sugar did not lead to the development of metabolic changes that characterize obesity. On the other hand, offering a diet with an excessive amount of fat leads to an increase in the adiposity index and visceral and body fat gain in comparison with sugar or control diets [3].

In addition, some studies highlighted that in their high-fat diets the main lipid source was saturated fatty acids [2, 3, 8–10, 16, 17, 21–30], while others did not discuss the type of fatty acids used. This information should be available in the articles, since quantity and quality of fatty acids can interfere in the success of obesity induction [8, 10]. Depending on the amount consumed, saturated or long-chain fatty acids can lead to a greater accumulation of body fat through the resynthesis of new triglycerides [27], as well as an increase in the production of inflammatory cytokines, which is a classical change observed in human obesity [27, 31].

The degree of response to the diet depends on its nutritional composition [11]. Additionally, the determination of nutritional composition is important to assess the occurrence of obesity and to evaluate the results considered control/standard. Therefore, control diets must have a nutritional basis similar to obesogenic diets, which helps to interpret the results without bias [8]. That is, the test diet and the control diet should differ only in relation to the specific macronutrient (carbohydrate or fat) used to induce obesity. The micronutrients, fiber and other ingredients must remain the same in order to observe how a specific macronutrient influences or not the outcome of obesity.

The palatability of diets interferes in the amount consumed. Therefore, the consumption of palatable diets (as cafeteria diets) is relevant to the increase in food consumption, including compulsive behavior, and consequently weight gain [8, 15]. Diets rich in salt, sugar and fat are known to have good palatability. The exposure to this type of diet interrupts the expression of clock-genes, modifying the day-night pattern of food intake, as well as changing dopamine signaling [15], which contributes to weight gain.

Caloric excess is essential for the development of obesity [19]. In this sense, although high-fat diets have a high sacietogenic potential, which reduces food consumption, the consumption of a small amount is able to efficiently increase weight and body fat due to the high caloric intake [7].

The diet-offering method directly affects consumption and the ability to induce obesity. Thus, it is likely that when the diet is offered according to the ad libitum or free access methods, food intake is stimulated [19]. On the other side, although the pair-feeding method limits the amount of diet to which animals will have access to, there are cases in which pair-fed diets can achieve different weight gain outcomes, something that has been attributed to the differences in macronutrient composition [32]. In addition, the amount of calories available may be different between groups, which allow high-calorie (obesogenic) diets to have the expected effect when compared to normocaloric diets, regardless of whether they have different palatability and satiety.

The intervention time required for the development of obesity varies widely, ranging from 8 days to 27 weeks (Table 2). Obesity phenotype [3, 30, 33–35], as well as metabolic changes typical of obesity–such as increased glucose intolerance–, [30] becomes more apparent after a longer exposure to an obesogenic diet. According to Blancas-Velasquez et al. [15], after 3 weeks of intervention there is a change in the pattern of fat consumption, which indicates that interventions lasting over 3 weeks may generate better results for the induction of obesity. Matias et al. [3] highlights that the seventh week was a turning point for the increase in weight gain; and Savetsky et al. [34] discussed the importance of a long intervention period, from 10 to 12 weeks, for the consolidation of the phenotypic and metabolic characteristics of obesity.

The consumption of a high-fat diet leads to changes in the composition of the intestinal microbiota [8], which is a classic parameter that usually accompanies the development of obesity [33, 36, 37]. The Western pattern diet, rich in sugar, fat and ultra-processed foods leads to changes in intestinal permeability, which results in an increase in endotoxemia, insulin resistance, steatosis and inflammation of the adipose tissue [38, 39], which results in obesity development [36, 38, 39].

Furthermore, obesity associated intestinal dysbiosis is characterized by a low microbial diversity and an imbalance between the different microorganisms of the intestinal microbiota, with a large number of pathogenic bacteria [8, 36, 39]. In this scenario there is lower production of short-chain fatty acids (SCFA, like acetate, propionate and butyrate) which leads to less protection of the intestinal epithelium, since SCFA are related to occludin and zonulin, and also leads to a drop in the production of glucagon-like peptide-1 (GLP-1) resulting in decreased satiety and increased insulin resistance, inflammation and lipid accumulation [24, 38, 40, 41].

Dysbiosis can also stimulate an excessive production of acetate, which can also contribute to the occurrence of obesity. This scenario occurs since the increase in acetate stimulates the activation of the parasympathetic pathway, which increases the secretion of ghrelin stimulating both an increase in food consumption and a greater secretion of insulin [42].

Therefore, we understand that one of the key factors for the development of obesity through the consumption of a high-fat diet is the alteration of the intestinal microbiota, always aiming for a state of eubiosis, that is, a balance in gut microbiota composition [33, 43].

### Experimental animals

The animal species most commonly used for obesity induction through diet is mouse, with isogenic or inbred strains, such as C57BL/6, C57BL/6J, AKR/J, and A/J [7]. In the present study, most of the analyzed studies used C57BL/6J mice (Table 2). Those animals are more susceptible to fat accumulation, gaining body weight and disruptions in glucose metabolism when fed an obesogenic diet [44]. Other strain used was the C57BL/6NCrl, which was developed for the study of lipoprotein and cholesterol metabolism (The Jackson Laboratory^©^) [45, 46].

Rats are used in DIO studies (Table 2). Sprague–Dawley rats are considered a good model for inducing obesity through diet, since they have a behavior similar to humans with regard to excessive food consumption, which can cause weight gain and changes in lipid metabolism [22]; however, Wistar rats are more susceptible to the development of obesity through diet, since they usually consume a higher amount of high-fat diet than the Sprague–Dawley. Also, differences in lipid metabolism, as fatty acid uptake and lipogenesis, as well as the interaction between genes and diet, make Wistar rats more susceptible to DIO [19].

The age [1] and the sex [47] of the animals can interfere in the development of obesity. Krishna et al. [1] observed that DIO develops better in younger animals. Additionally, when young, speed in weight gain is also greater because elderly animals can adapt metabolically to the increase in adiposity; also, less inflammation is observed in these animals, which causes less glycemic and hepatic alterations [1]. Mice aged 6 to 8 weeks can be considered young adult mice [15].

Young male mice have bigger weight gain than females; however, when they are middle-aged the opposite occurs, and female mice have bigger weight gain than males. Inflammatory genes—upregulated in juvenile males—and hormonal parameters—estrogens can regulate inflammatory pathways in female—may justify the results found [47]. Gene expression in the arcuate nucleus—low in males—[48] and differences in metabolic programming between males and females—related to the expression of genes in the endoplasmic reticulum and hepatic energy metabolism– also contribute to sex-specific weight gain [49].

Male animals are commonly used in the study of obesity; however, if the study aims to evaluate the brown adipose tissue, females should be used, since this tissue is more easily observed in this sex [50, 51]. However, as a limitation that currently exists, it is important to find a model that achieve a similar obesity degree in both, males and females, in order to study both sexes in the same experiment.

### Main parameters used to assess the development of obesity

#### Food intake

Calorie overconsumption leads to an increase in body weight gain and abdominal fat accumulation [2, 10, 15, 19]. These body alterations may lead to deactivation of liver and mesenteric genes responsible for beta-oxidation [25]. Besides, it can deregulate AMPK [2, 35] and SIRT1 proteins in the mesenteric white adipose tissue and skeletal muscle [2]. Additionally, the increase in abdominal fat accumulation can raise blood leptin concentrations [3, 15], leading to leptin resistance. Thus, both the increase in weight and body fat mass generate a cycle that feeds back on itself.

The taste and texture of diets influence the amount of food consumed. Diets rich in processed foods, with high levels of sodium, sugar and saturated fatty acids, are more palatable, which can lead to a higher weight gain in comparison with purified diets even when saturated fatty acids are added to them [52]. Additionally, DIO must contain a low concentration of fibers [25], since these nutrients are capable to induce satiety and increase the production of GLP-1 and SCFA, which stimulate a lower energy consumption [24].

Considering that a spontaneous caloric increase is difficult to achieve in rodents, even when flavored diets are offered [6], DIO must have a high caloric density [16, 30].

In an attempt to induce a voluntary hyperphagia, Blancas-Velazquez et al. [15] provided extra calories to C57BL/6J mice through a 10% water-sugar solution (0.4 kcal/ml). With the sugar solution, the animals had free access to regular chow food, fat-rich pellets, and a bottle of tap water. In this way, the mice could eat what pleased them most, allowing hyperphagia and obesity induction [15]. The problem is that it is difficult to calculate the amount of calories that each animal consumes.

#### Markers related to body weight and adiposity

Because of the absence of a specific marker and a consensus, for both mice and rats, that defines the presence or absence of obesity, some studies have established their own parameters: the difference of 15% [53] or 20 g in body weight between test and control groups [54]; adiposity index determination [3]; creation of cutoff points [1]; and calculation of body mass index [55].

No article considered the distribution of body composition (calculated by DXA or EchoMRI) as a parameter for detecting obesity. However, the majority of studies consider the differences in total body weight gain as the main parameter to assess the outcome of the development of obesity (Table 3).

**Table 3:** Changes in markers related to body weight and adipose tissue depots

Article ID	Weight gain	Final body weight	Total adipose tissue/ Body fat	White adipose tissue	Mesenteric fat	Epididymal fat	Perirenal fat	Brown adipose tissue	Lee index
Asokan et al. 2018 [56]	–	HF = Standard	HF = Standard	–	–	–	–	–	–
Bortolin et al. 2018 [8]	HF = Control	HF = Control	–	HF = Control	–	–	–	–	–
	WD > Control	WD > Control	–	WD > Control	–	–	–	–	–
Heo et al. 2018 [2]	HF > LF	HF > LF	–	HF > LF	HF > LF	HF > LF	HF > LF ○	HF = LF	–
Hira et al. 2018 [24] (For 8 days and/or 8 weeks)	HFSuc > Control	HFSuc > Control	–	–	–	–	–	–	–
Iñiguez et al. 2018 [25]	HF > Control	–	HF > Control	–	HF > Control	HF > Control	–	–	–
Kazeminasab et al. 2018 [12]	HF > LF	–	–	–	–	–	–	–	–
Lee et al. 2018 [27]	All HF > All LF	All HF > All LF	All HF > All LF	–	All HF > All LF	All HF > All LF	All HF > All LF	–	–
Matias et al. 2018 [3]	–	HF > Control	HF > Control	–	–	–	–	–	–
	–	HS = Control	HS = Control	–	–	–	–	–	–
	–	HFHS = Control	HFHS > Control	–	–	–	–	–	–
Miranda et al. 2018 [19]	–	HF SD = Control SD	HF SD = Control SD	–	HF SD = Control SD	HF SD > Control SD	HF SD = Control SD	–	–
	–	HF W > Control W	HF W > Control W	–	HF W > Control W	HF W > Control W	HF W > Control W	–	–
	–	HF > Control	HF > Control	–	HF > Control	HF > Control	HF > Control	–	–
Rocha Rodrigues et al. 2018 [57]	–	HF = Standard	–	–	–	–	–	–	HF = Standard
Son et al. 2018 [21]	HF > Normal	–	HF > Normal	–	HF > Normal	HF > Normal	HF > Normal	–	–
Virto et al. 2018 [62]	–	HF > Control	–	–	–	–	–	–	–
Wu et al. 2018 [30]	–	HF > Normal	–	–	–	–	–	–	–
Yuan et al. 2018 [35] 18 semanas	–	HF = Normal	–	–	–	–	–	–	–
Yuan et al. 2018 [35] 24 semanas	–	HF > Normal	HF > Normal	HF > Normal	–	HF > Normal	HF > Normal	–	–
Aslani et al. 2017 [63]	–	HF > Normal	HF > Normal	–	–	–	–	–	HF > Normal
Blancas-Velazquez et al. 2017 [15]	–	HFHS > Control	–	–	–	–	–	–	–
Karimi et al. 2017 [18] 12 semanas	–	HF > Standard	–	–	–	–	–	–	–
Karimi et al. 2017 [18] 27 semanas	HF = Standard	HF > Standard	HF > Standard	–	–	–	–	–	–
Kim et al. 2017 [33]	–	HF > Standard	–	–	–	–	–	–	–
Kus et al. 2017 [68]	–	HF > Control	–	–	–	–	–	–	–
La Frano et al. 2017 [26]	–	HFHC > LFLC	–	–	–	HFHC > LFLC	–	–	–
Pan et al. 2017 [61]	HF > Normal	HF > Normal	–	–	HF > Normal	–	–	–	–
Picklo et al. 2017 [10]	–	HFO > LF	HFO > LF	–	–	HFO > LF	–	–	–
	–	HFSat > LF	HFSat > LF	–	–	HFSat > LF	–	–	–
Yang et al. 2017 [22]	–	HF > Control	–	–	–	–	–	–	HF > Control
Choi et al. 2016 [9]	HF > Normal	HF > Normal	HF > Normal	–	HF > Normal	HF > Normal	HF > Normal	–	–
Jambocus et al. 2016 [17]	HSF > Normal	–	–	–	–	–	–	–	–
Krishna et al. 2016 [1]	–	HF > Standard	HF > Standard	–	–	–	–	–	–
Naidu et al. 2016 [20]	–	HF > Normal	–	–	–	–	–	–	–
Zhao et al. 2016 [64]	HF > Control	HF > Control	–	–	–	HF > Control	HF > Control	–	–
Huang et al. 2015 [16]	HF > Normal	HF > Normal	–	–	–	HF > Normal	HF > Normal	–	–
Li et al. 2015 [51]	–	HF > Control	–	HF > Control	–	–	–	HF > Control	–
Nam et al. 2015 [28]	–	HF > LF	–	–	–	HF > LF	HF > LF	–	–
Rodríguez- Rodríguez et al. 2015 [29]	–	HF > Control	–	–	–	–	–	–	–
Savetsky et al. 2015 [34]	–	HF > Normal	–	–	–	–	–	–	–
Wyatt et al. 2015 [13] (For 5 and 7 weeks)	–	HF > Control	HF > Control	–	–	–	–	–	–
Zhang et al. 2015 [23]	–	HF > Normal	–	HF > Normal	HF = Normal	HF > Normal	–	–	–

HF, High-fat diet; HFHC, High-fat high-cholesterol diet; HFHS, High-fat high-sugar diet; HFSat, High-fat saturated diet; HFSuc, High-fat high-sucrose diet; HFO, High-fat oleic diet; HS, High-sugar diet; LF, Low-fat diet; LFLC, Low-fat low-cholesterol diet; SD, Sprague–Dawley; W, Wistar; WD, Western diet; ○, Perirenal + Retroperitonial

When there is no significant difference in body weight gain, other parameters can be considered [3, 56, 57]. In this way, Matias et al. [3] did not observe differences in weight gain after the animals consumed a high-fat/high-sugar diet, but there was a gain in total white adipose tissue, which indicates the occurrence of obesity. Also, Rocha-Rodrigues et al. [57] reported an increase in visceral adipose fat compared to weight gain, as well as an increase in leptin concentration in animals fed a high-fat diet.

Visceral fat is the depot that surrounds the abdominal organs. It is more vascularized, innervated, inflammatory, metabolically active and sensitive to lipolysis, which results in greater release of cytokines, fatty acids and triglycerides. Therefore, in humans it is related to a higher mortality prediction when compared to subcutaneous adipose fat [58, 59]. In Wistar rats the consumption of a high-fat diet appears to lead to an increase in the number of fat cells (hyperplasia) in the subcutaneous adipose fat, whereas in the visceral adipose fat greater hypertrophy of the adipose tissue is observed [60].

Body fat accumulation depends on the connection between different metabolic pathways, as well as the interaction between genes and diet. Wistar rats, for example, have a differential expression of genes in the subcutaneous adipose tissue in comparison with Sprague–Dawley rats, which justifies the higher fat depots found in this breed [19]. The consumption of a high-fat diet leads to an increase in the uptake of fatty acids and lipogenesis [19, 33], resulting in adipocytes hyperplasia [2, 20, 61] and hypertrophy [2, 20, 28, 35, 61]. This increase in adipose tissue can cause tissue hypoxia, which can impair the production and release of obesity regulatory hormones, such as leptin, adiponectin and ghrelin, and exacerbate inflammation [57]. Additionally, body fat increase can cause muscular cell damage, since it enhances cell susceptibility to protein degradation and apoptosis [56]; therefore, DIO is able to cause the metabolic and morphological changes that characterize human obesity.

A low-grade inflammatory condition is often observed in obese animals [1, 2, 27, 28, 34, 35, 56]. This inflammatory status can be triggered by a high consumption of saturated fatty acids, which can be found in high concentrations in obesogenic diets [27]. Diets rich in saturated fats can elevate the production of inflammatory cytokines, such as TNF-α [1, 2, 28, 35, 56] and IL-6 [56], as a consequence of the hypertrophy of the adipocytes [2, 35], leading to an infiltration of macrophages and dendritic cells in adipose tissue [1].

This inflammatory condition contributes to the development of metabolic disorders [1, 35], such as diabetes [27]; to a decrease in lymphatic function and cutaneous hypersensitivity [34]; and to the occurrence of other diseases, such as periodontitis [62] and respiratory allergies [63]. Furthermore, the immune system can also be altered, with an improvement in this system when there is a modulation of the production of inflammatory cytokines [64].

The consumption of a high-fat diet can also reduce the brown adipose tissue, since it may inhibit the biosynthesis of fatty acids and increase oxidative stress and cell apoptosis [51]; therefore, a high-fat diet can stimulate the development of white adipose tissue [2, 8–10, 16, 19, 21, 23, 25–28, 35, 51, 61, 64] and suppress the development of the brown adipose tissue [51]. This is relevant since the brown adipose tissue, in humans, is negatively correlated with the body mass index and with central obesity markers which suggests that low levels of brown adipose tissue may be indicator of obesity and obesity-related diseases [65].

In addition, the increase in brown adipose tissue may be a strategy to fight obesity, since higher levels of brown adipose tissue indicate greater energy expenditure, with consequent weight loss [65, 66]. Therefore, low levels of brown adipose tissue can contribute to the perpetuation of obesity.

#### Glycemic markers

Higher values in blood glucose [8, 9, 16, 17, 20, 23, 27, 29, 51] and insulin concentrations [1, 2, 8, 9, 16, 17, 20, 23, 25–27, 29] as well as in HOMA (homeostatic model assessment) index [2, 8, 23–25, 27, 29, 57] have been observed in the groups that consumed an obesogenic diet. Similarly to obesity, for an effective induction of insulin resistance, a long period of intervention with DIO must occur [25]. In this sense, the absence of changes in these parameters in some studies can be justified by the short intervention period (Table 2).

Changes in the glycemic parameters occur because the insulin metabolism is unable to adapt to the damage caused by the chronic excess of calories offered by the obesogenic diet, which gradually deteriorates insulin activity, leading to insulin resistance and subsequent type 2 diabetes development [67]. Also, the obesogenic diet can induce fat (ectopic) accumulation in the pancreas, which stresses greatly beta cells, disrupting insulin production [23, 27]. This accumulation in organs other than the adipose tissue, such as the liver, can also lead to insulin resistance and hyperglycemia, since saturated fatty acids interfere in the activity of insulin receptor and glucose transporters [23]. Additionally, the mitochondria of the brown adipose tissue are also affect by DIO impairing glucose metabolism [51].

#### Serum lipid profile

Triglycerides are the main component of adipose tissue; therefore, high serum concentrations of this lipid may ​​indicate the presence of metabolic changes [21]. Likewise, serum concentration of cholesterol is also an important parameter for the assessment of obesity, since the greater the availability of serum cholesterol, the greater the deposition of fatty acids into the adipose tissue and the liver [33].

In this way, some of the evaluated studies noticed an increase in the serum concentration of triglycerides [1, 2, 9, 16, 17, 21–23, 25, 35, 51, 61, 63] and cholesterol [2, 9, 16, 23, 27, 29, 35, 51, 61, 63] in the groups fed with DIO. This change seems to be especially related to saturated fatty acid-rich [27] obesogenic diets (Table 2). Additionally, diets with high concentration of long-chain fatty acids can also alter the serum lipid profile, since, after hydrolysis, these fatty acids can be used for the synthesis of new triacylglycerol molecules [27].

#### Liver health

Liver health, measured through hepatic triglycerides, can be impaired by the development of obesity. Hepatic steatosis happens because the excess of fat present in the body is stored in this organ causing intracytoplasmatic accumulation of triglycerides. This ectopic accumulation occurs as a consequence of the downregulation of AMPK [35, 61] and upregulation of SREBP-1c [61], which generates lipogenesis, and increases the synthesis of fatty acids by the liver [23]. Furthermore, beta-oxidation is downregulated, which increases the hepatic lipid stocks [16, 23, 25]. Thus, the reduction in this parameter is positive for the treatment of obesity, with the expression of lipogenic genes conditioned [21].

As the amount of stored fat increases, the liver starts to suffer oxidative damage [16] and, as a result of hepatocyte lysis, the serum concentrations of the enzymes alanine aminotransferase and aspartate aminotransferase increase [16, 21, 68]. Thus, the increase in the concentrations of both aminotransferases can be associated with the increase in liver weight as well as to hepatic steatosis [25]. In this context, it has been described that high-fat diet (71% of kcal fat) induces similar degree and pattern of steatosis and liver triglyceride content in male Wistar and Sprague–Dawley rats, being males more susceptible than females [69].

## Conclusions

In the present study, we included studies that used Sprague–Dawley and Wistar rats, as well as C57BL-/-6 mice, because these are the main models used for DIO. This strategy can be considered a limitation of the study, since other rodent models may also be prone to diet-induced obesity; however, they are not widely used. In fact, the biggest difficulty to find an effective model for DIO is the lack of standardization among obesity-inducing protocols. Different times of intervention, diets, types of fat and carbohydrates, animal strains, and sex, among others, are used in the studies, which makes it difficult to compare the results and to better evaluate and determine the best way to induce obesity in an animal model.

Among the animal obesity models, those that develop a phenotype more similar to human physiopathology are those induced by dietary challenge; in this context, better results are obtained through high-fat diets with high concentrations of saturated fatty acids, since these diets directly affects the metabolism, are palatable and have a high caloric density, which stimulates weight and body fat gain.

To choose an animal model for a study of diet-induced obesity, it should be considered that rats and mice respond differently to this type of diet; in addition, strain, sex and age, affect the response to the obesogenic diet, with young animals and males being more sensitive to obesity-related comorbidities.

The markers used to assess the development of obesity include body weight and fat (total, subcutaneous and visceral) gain, but other parameters related to inflammation, hormone concentration, blood glycemia, lipid profile, and liver health are often desired. It is suggested that these markers should be used together, since the presence of more than one of these markers reinforces the determination of obesity. Changes in the release of inflammatory cytokines are used to justify the symptoms found, not being a determining parameter for the induction of obesity. As there are no cutoff points for any of these parameters in animals, researchers should always conduct their studies with a non-obese control group so that the results can be compared.

## Data Availability

Not applicable.

## Abbreviations

DIO: Diet induced-obesity
DXA: Dual energy X-ray absorptiometry
GLP-1: Glucagon-like peptide-1
SCFA: Short chain fatty acids
